# The Mixed-Lineage Kinase Inhibitor URMC-099 Protects Hippocampal Synapses in Experimental Autoimmune Encephalomyelitis

**DOI:** 10.1523/ENEURO.0245-18.2018

**Published:** 2018-12-03

**Authors:** Matthew J. Bellizzi, Jennetta W. Hammond, Herman Li, Mary A. Gantz Marker, Daniel F. Marker, Robert S. Freeman, Harris A. Gelbard

**Affiliations:** 1 Center for Neurotherapeutics Discovery, University of Rochester Medical Center, Rochester, NY 14642; 2 Department of Neurology, University of Rochester Medical Center, Rochester, NY 14642; 3 Department of Neuroscience, University of Rochester Medical Center, Rochester, NY 14642; 4 Department of Pharmacology and Physiology, University of Rochester Medical Center, Rochester, NY 14642; 5 Departments of Pediatrics and Microbiology and Immunology, University of Rochester Medical Center, Rochester, NY 14642

**Keywords:** microglia, multiple sclerosis, neurodegeneration, neuroprotection, synapse

## Abstract

Treatments to stop gray matter degeneration are needed to prevent progressive disability in multiple sclerosis (MS). We tested whether inhibiting mixed-lineage kinases (MLKs), which can drive inflammatory microglial activation and neuronal degeneration, could protect hippocampal synapses in C57BL/6 mice with experimental autoimmune encephalomyelitis (EAE), a disease model that recapitulates the excitatory synaptic injury that occurs widely within the gray matter in MS. URMC-099, a broad spectrum MLK inhibitor with additional activity against leucine-rich repeat kinase 2 (LRRK2) and other kinases, prevented loss of PSD95-positive postsynaptic structures, shifted activated microglia toward a less inflammatory phenotype, and reversed deficits in hippocampal-dependent contextual fear conditioning in EAE mice when administered after the onset of motor symptoms. A narrow spectrum inhibitor designed to be highly selective for MLK3 failed to protect synapses in EAE hippocampi, and could not rescue cultured neurons from trophic deprivation in an *in vitro* model of MLK-driven neuronal degeneration. These results suggest that URMC-099 may have potential as a neuroprotective treatment in MS and demonstrate that a broad spectrum of inhibition against a combination of MLK and other kinases is more effective in neuroinflammatory disease than selectively targeting a single kinase.

## Significance Statement

Multiple sclerosis (MS) causes progressive neurologic decline that correlates with damage to gray matter in the brain, and is not stopped by current treatments. We used a mouse model of MS, in which gray matter injury is associated with inflammatory molecules produced by activated microglia, to test novel neuroprotective treatments. URMC-099, a mixed-lineage kinase (MLK) inhibitor that has anti-inflammatory and direct neuroprotective properties, prevented loss of hippocampal synapses and reversed deficits in learning and memory in a mouse model of MS. An MLK inhibitor with a narrower range of specificity was not effective, suggesting that combination kinase inhibition with URMC-099 may have potential as a neuroprotective treatment in MS.

## Introduction

Preventing gray matter degeneration is an important goal in the treatment of multiple sclerosis (MS). Gray matter injury, measured by atrophy in neuroimaging studies, can begin early in the course of disease and progress in parallel with worsening physical and cognitive disability in MS patients (Dalton et al., 2004; Fisniku et al., 2008).

While demyelinated gray matter lesions have been associated with neuronal damage and loss of synapses (Peterson et al., 2001; Wegner et al., 2006; Dutta et al., 2011), neuronal injury occurs in myelinated “normal-appearing” gray matter (NAGM) as well and mirrors patterns of microglial activation within and outside of cortical lesions (Magliozzi et al., 2010). Injury to excitatory synapses with loss of postsynaptic dendritic spines has been found throughout the cortex at similar rates within demyelinated lesions and NAGM (Jürgens et al., 2016). Occurring independently of local demyelination or axon loss, this synaptic injury may underlie the disability that can progress out of proportion to lesion burden and despite current immunomodulatory treatments (Roosendaal et al., 2011; Hauser et al., 2017).

Similar synaptic injury occurs in the hippocampus of mice with experimental autoimmune encephalomyelitis (EAE), providing a model for studying the pathogenesis and treatment of synaptic injury in MS gray matter. Damage to excitatory postsynaptic structures in EAE is mediated by platelet-activating factor (PAF), a signaling molecule that can be released downstream of microglial activation (Bellizzi et al., 2016). Inflammatory microglia also release tumor-necrosis factor-α (TNFα) and interleukin-1β (IL-1β), which have been found to promote neuronal injury in other models of MS gray matter injury (Centonze et al., 2009; Rossi et al., 2012).

Inflammatory molecules can be difficult to target therapeutically due to the redundancy of multiple mediators activated in parallel and because many of these molecules play critical signaling roles in synaptic physiology as well as in pathology. Blockade of PAF signaling could prevent synapse loss in the EAE hippocampus (Bellizzi et al., 2016) but also disrupts learning and memory (Jerusalinsky et al., 1994), and treatments targeting TNFα had deleterious effects in MS clinical trials (van Oosten et al., 1996).

While microglia can produce molecules with neurotoxic effects, they can also become activated to promote neuroprotection and repair in inflammatory disease (Chen and Trapp, 2016). Shifting activated microglia toward a protective phenotype may provide an effective strategy for gray matter neuroprotection in MS. The phosphodiesterase type 4 inhibitor, ibudilast, which can reduce proinflammatory microglial activation *in vitro*, has slowed rates of brain atrophy in early-phase clinical trials (Barkhof et al., 2010; Fox et al., 2018).

Inhibition of mixed-lineage kinases (MLKs) has potential to both modulate microglial phenotype and directly protect neurons against degeneration. MLKs are activated in response to inflammation and cell stress, and mediate neuronal degeneration (Harris et al., 2002; Tian et al., 2003; Lotharius et al., 2005) and proinflammatory activation of macrophages and microglia (Ciallella et al., 2005; Sui et al., 2006; Eggert et al., 2010) via c-Jun N-terminal kinase (JNK) and other MAP kinase pathways. MLK3, which is widely expressed and can link glutamatergic stimulation to JNK activation at excitatory synapses (Savinainen et al., 2001), has been a target of interest for treatment of several neurodegenerative diseases (Maroney et al., 2001; Goodfellow et al., 2013).

URMC-099 is a brain-penetrant small molecule designed to inhibit MLK3 that has attenuated proinflammatory microglial activation and protected against neuronal and synaptic damage in models of neuroinflammatory disease *in vitro* and *in vivo* (Marker et al., 2013; Dong et al., 2016; Kiyota et al., 2018). Here, we investigated whether URMC-099 treatment could protect synapses and hippocampal function in mice with EAE. Because URMC-099 has potent inhibitory activity against several MLKs as well as other kinases including leucine-rich repeat kinase 2 (LRRK2; Goodfellow et al., 2013), we additionally compared URMC-099 against CLFB_1134_, a highly-specific MLK3 inhibitor with a different chemical scaffold but also with an excellent CNS pharmacokinetic profile (Goodfellow et al., 2016), to determine whether narrow versus broad specificity could enhance neuroprotection in the EAE hippocampus.

## Materials and Methods

### Animals

Male and female C57BL/6 mice were purchased at age seven weeks (The Jackson Laboratory, catalog #JAX:000664, RRID:IMSR_JAX:000664) and were vivarium-housed for at least one week before EAE induction. All animal experiments were performed in accordance with the University of Rochester University Committee on Animal Resources according to the Public Health Service Policy on Humane Care and Use of Laboratory Animals.

### EAE

We immunized mice of either sex with pre-mixed emulsion (Hooke Labs, catalog #EK-2110) containing myelin oligodendrocyte glycoprotein peptide amino acids 35–55 (MOG_35–55_), in complete Freund’s adjuvant (CFA) containing heat-inactivated mycobacterium tuberculosis H37RA. 100 µl of emulsion was injected subcutaneously at each of two sites over the upper and lower back. Sham-immunized sex-matched littermate controls were injected with control emulsion with MOG_35–55_ omitted (Hooke Labs, catalog #CK-21100), and otherwise received identical treatment. Mice were injected intraperitoneally with pertussis toxin (Hooke Labs, 90–400 ng, with the dose adjusted according to potency of each lot per manufacturer recommendations) on 0 and 1 d post-immunization (dpi). Mice were subsequently monitored for signs of clinical disease and scored for motor deficits as follows: 0, no deficit; 0.5, partial tail paralysis; 1, complete tail paralysis; 2, hindlimb weakness; 2.5, paralysis of one hindlimb; 3, paralysis of both hind limbs; 3.5, hindlimb paralysis and forelimb weakness; 4, quadriplegia; 5, moribund. Scores are reported relative to day of disease onset (day 0: first score 0.5 or greater) when drug treatment was begun.

### Drug treatment

For *in vivo* experiments, URMC-099 (Califia Bio, Inc., and Pharmaron) and the highly-selective MLK3 inhibitor CLFB_1134_ (Califia Bio, Inc.) were dissolved at 2 mg/ml in a sterile solution of 5% DMSO, 40% polyethylene glycol 400, and 55% normal saline and stored at room temperature protected from light. Mice were randomized to drug treatment beginning on the first day of motor deficits (EAE score 0.5 or greater). Randomization was stratified for timing and severity of symptom onset, and sham-immunized mice were assigned to begin treatment at the same time. The initial cohort of mice was randomized to treatment with URMC-099 (10 mg/kg, i.p., twice daily), CLFB_1134_ (30 mg/kg, i.p., twice daily), or vehicle control (all mice received identical volumes of vehicle per weight). Dose selection of URMC-099 and CLFB_1134_ was based on affinity for MLK3 and prior studies of CNS pharmacokinetics for URMC-099 and similar compounds (Goodfellow et al., 2013), as well as demonstrated *in vivo* effects of URMC-099 in other models of neuroinflammation (Marker et al., 2013). After establishing the relative efficacy of URMC-099 versus CLFB_1134_ for protection of hippocampal synapses, subsequent cohorts were treated with URMC-099 or vehicle only. Experimenters were blinded to treatment assignment throughout each experiment and data analysis.

### Cued and contextual fear conditioning

Mice underwent fear conditioning 12–14 d after onset of EAE symptoms and drug treatment, by which time mice were acclimated to human handling after daily EAE scoring and injections. For conditioning, we transferred mice individually to an isolation chamber (Coulbourn Habitest H10-24T) with a 7” × 7” square test cage with metal walls and a grid floor of 16 stainless steel bars that were wired to deliver an electric shock. We inserted a Plexiglas plate to support and facilitate mobility for mice with paretic limbs who could not maintain an ambulatory posture on the bars. After 3 min in the chamber, mice were presented with a 15-s white noise cue coterminating with a 2-s shock (0.55 mAmps delivered through the grid floor via a Coulbourn precision animal shocker). Paired cue and shock were delivered three times, 30 s apart. Mice were removed from the chamber to a holding cage until all of their littermates had completed the conditioning protocol, and then returned to their home cage overnight. The chamber floor was removed, wiped with water, and dried between each trial. Twenty-four hours later, we tested contextual conditioning responses by returning mice to the same chamber (prepared identically as for conditioning) for 5 min without stimulus (conditioned context test). We recorded video via a camera fixed directly above the cage for subsequent motion analysis. After returning mice to their home cage (via a holding cage until littermates had completed testing) for 3 h, we tested cued conditioning responses. We placed mice in a round test cage with smooth plastic walls and floor that was wiped with ethanol before each trial, lined on the floor with clean cage bedding, and illuminated in the isolation chamber with a red light to provide a novel context that differed from the conditioned context in shape, texture, odor and color. We recorded video for freezing analysis for 3 min without stimulus (novel context test) followed by 3 min of continuous presentation of the white noise cue (cue test). For each test, mice were placed in the test chambers in the same order which was randomized with respect to treatment groups, with males run before females.

We measured contextual and cued fear conditioning responses using motion analysis software (Freeze Frame/Freeze View, Actimetrics Software) to detect episodes of freezing behavior, which were defined as ≥0.5-s bouts of immobility except for breathing, in which the software-generated motion index was below a threshold value that was confirmed via video review for each mouse to discriminate freezing from exploratory, grooming, sniffing or rearing behaviors. We quantified freezing responses as the percentage of total time spent freezing during the first 3 min in the conditioned context, 3 min in the novel context, and the first 90 s of the cue test (beyond which responses tended to deteriorate at variable rates). Because EAE mice had increased rates of immobility at baseline and across all conditions, reflecting motor deficits and/or decreased exploratory behavior and non-specific to context or cue, we subtracted rates of freezing during exposure to the novel, unconditioned context from rates during the conditioned context and tone tests to generate a measure (Δ freeze) that reflected increased freezing due to the mouse’s ability to discriminate between the conditioned and novel context, and in response to the cue. These values were normalized to those of the sham-immunized controls for the same testing cohort, to account for variations in the magnitude of the conditioning effect among cohorts. Mice with >60% immobility in the unconditioned novel context (4/22 mice in the EAE-vehicle group and 5/26 EAE-099 mice) were excluded from the Δ freeze analysis as high baseline immobility obscured the ability to measure conditioning effects; none of the remaining mice had mean freezing rates of >31% in the novel context.

### Immunohistochemistry

After 12–14 d of treatment, mice were anesthetized with ketamine/xylazine (200 and 1 mg/kg, respectively) and intracardially perfused for 1 min with PBS containing EDTA (1.5 mg/ml) followed by 4% paraformaldehyde (PFA; 100 ml perfused over 30 min). Brains were removed, post-fixed for 24 h in 4% PFA and stored at in PBS at 4°C; 40-µm coronal sections were cut on a vibratome (Leica V1000) and stored in cryoprotectant (30% PEG300, 30% glycerol, 20% 0.1 M phosphate buffer, and 20% H_2_O) at –20°C. Free-floating sections were washed in PBS (3 × 30 min) followed by glycine (100 mM) to reduce autofluorescence, incubated in citrate antigen unmasking solution (Vector H3300) with 0.05% Tween for 30 min at 37° C, and washed again in PBS. We incubated sections for 2 d at room temperature in 1.5% BSA (Sigma), 3% normal goat serum (Vector Laboratories), 0.5% Triton X-100 (Promega), and 1.8% NaCl for blocking and permeabilization, mixed with the following primary antibodies: mouse anti-PSD95 (NeuroMab, catalog #75-028, RRID:AB_2307331; 1:500), rabbit anti-Iba1 (Wako Biochemicals, catalog #019-19741, RRID:AB_839504; 1:1000), rabbit anti-Tmem119 (Abcam, catalog #ab209064, RRID:AB_2728083, 1:200), chicken anti-Iba1 (Synaptic Systems, catalog #234 006, RRID:AB_2619949; 1:500), rat anti-CD68 (Abd Serotec, catalog #MCA1957, RRID:AB_322219; 1:1000), rat anti-Ly-6B (clone 7/4, Abcam, catalog #ab53457, RRID:AB_881409; 1:1000), or mouse anti-MAP2 (Sigma, catalog #M4403, RRID:AB_477193; 1:500). We washed sections in PBS with 1.8% NaCl (3 × 30 min) and then incubated overnight at room temperature with Alexa Fluor-conjugated secondary antibodies (Invitrogen, 1:500) in the same blocking/permeabilization mixture as above. After washing in PBS (3 × 30 min) we mounted sections on slides with Prolong Gold mounting agent (Life Technologies) and #1.5 cover glass.

### Tissue section imaging and analysis

Slides were coded during image collection and analysis to blind the experimenters to treatment condition. We imaged tissue sections by grid pseudo-confocal microscopy, using an Olympus BX-51 microscope equipped with Quioptic Optigrid hardware for optical sectioning and a Hamamatsu ORCA-ER camera, controlled by Volocity 3DM software (PerkinElmer Life and Analytical Science). Differences in *z*-axis registration of different fluors were corrected by calibration with multicolor fluorescent beads. We imaged the dorsal CA1 hippocampal area, collecting images from 6 sections per animal and using identical light intensity and exposure settings for all animals within each image set. We quantified density of postsynaptic puncta from 60× image stacks (z-step = 0.3 µm) of PSD95 staining in the CA1 stratum radiatum, using a custom-designed automated algorithm in Volocity software to identify puncta based on size and local intensity maxima (identified by both Find Objects and Find Spots algorithms). In the same imaging fields, we quantified microglial Iba1 intensity by the sum of Iba1 pixel values within Iba1-positive objects, and microglial CD68 by the proportional volume of Iba1 objects that were also positive for CD68. We measured the ratio of surface area to volume for Iba1-positive objects to quantify microglial changes from a ramified to more amoeboid morphology. For all measurements, values from image stacks in six tissue sections per mouse were averaged and expressed relative to sham-immunized controls from the same treatment cohort. Image stacks are displayed as two-dimensional extended-focus images collapsed along the *z*-axis.

### Hippocampal Western blottings

Mice at 29 dpi were perfused briefly with PBS and hippocampi were isolated, immediately frozen on dry ice, and stored at –80°C. Individual hippocampi were homogenized in RIPA lysis buffer containing a protease inhibitor cocktail (MilliporeSigma: Calbiochem, SetV). The cell lysate was kept on ice with periodic vortexing for 30 min, then centrifuged at 13,000 rpm for 10 min. The supernatant was centrifuged again at 13,000 rpm for 10 min. The protein concentration of the final supernatant lysate was assayed with a detergent-compatible Bradford assay (Pierce). Equivalent amounts of protein between samples were then mixed with loading dye and run on an 8% or 12% SDS-PAGE gel and transferred to PVDF. Membranes were blocked with 5% milk in tris-buffered saline (TBS) with 0.5% Tween-20 (TBST) for 30 min and probed with one of the following primary antibodies in 5% milk in TBST for 1–16 h: Iba1 (Wako Biochemicals, catalog #019-19741, RRID:AB_839504), TNFα (Cell Signaling Technologies, catalog #11948, RRID:AB_2687962), IL1β (Genzyme, catalog #1886-01), iNOS (Cell Signaling Technologies, catalog #2982S, RRID:AB_1078202), FcγR1/CD64 (R&D Systems, catalog #AF2074, RRID:AB_416550), CD86 (BD PharMingen, catalog #553689, RRID:AB_394991), actin (Santa Cruz, catalog #SC-47778, RRID:AB_626632), IL10 (AbD Serotec, catalog #MCA1302G, RRID:AB_2125093), Arg1 (Cell Signaling Technologies, catalog #93668), C1q [1151 (Huang et al., 1999), gift of Andrea Tenner]. Membranes were washed three times in TBST and then incubated with HRP secondary antibodies for 1 h in 5% milk in TBST. After washing we applied ECL substrate (Pierce) and developed membranes using a digital imager (Azure Biosystems). Membranes were stripped using buffer consisting of 200 mM glycine, 0.1% SDS, and 1% Tween 20 (pH 2.2) and re-probed up to four times. Western blotting band densities were quantitated using ImageJ. Band densities for each protein were first normalized to the corresponding actin band densities for each animal from the same gel then normalized to the control group mean.

### Primary neuronal cultures

Sympathetic neuron cultures were prepared from superior cervical ganglia (SCG) of newborn (postnatal day 0–1) male and female C57Bl/6J mice. SCG from individual mice were pooled and incubated at 37°C for 20 min in 2.5 mg/ml collagenase (Worthington Biochemical) followed by a second 20-min incubation in 1 mg/ml trypsin (Worthington Biochemical). The SCG were rinsed twice in culture media consisting of modified Eagle’s medium supplemented with 10% fetal bovine serum, 100 U/ml penicillin, 100 µg/ml streptomycin, 20 µM uridine, 20 µM fluorodeoxyuridine, 2 mM L-glutamine, and 50 ng/ml nerve growth factor (NGF; Harlan Products for Bioscience) and then triturated with a fire-polished Pasteur pipette to obtain a single cell suspension. Cells were diluted in fresh NGF-containing media and plated onto poly-L-lysine and laminin-coated glass slides (cell survival assays) or collagen-coated tissue culture dishes (Western blotting experiments). Cultures were maintained in a 5% CO_2_ incubator at 37°C for 5 d before initiating treatments. For NGF deprivation, neurons were rinsed twice in culture medium lacking NGF and then incubated in the same NGF-free media supplemented with sheep anti-NGF antiserum (Cedarlane Laboratories) to scavenge residual NGF. For treatment with URMC-099 or CLFB_1134_, we prepared 100-µm stock solutions of each drug in DMSO that were diluted to 100 or 300 nM final concentrations in culture media and added at the start of NGF deprivation.

### Cell survival assays

Just before initiating treatments, the number of healthy neurons in each of four pre-marked fields of view (>100 neurons per field of view) was determined using phase-contrast microscopy at 100× magnification. Neurons were scored as healthy if they exhibited a rounded and refractile cell body, a clearly defined nucleus and nucleolus, and smooth and intact neurites. At the end of the treatment period, neurons were fixed in 3% PFA for 20 min and then stained with the DNA-binding dye Hoechst 33 342 (Invitrogen). The number of healthy neurons in the same four pre-marked fields was once again determined based on the criteria described above plus the presence of diffuse Hoechst-stained chromatin completely filling the nucleus. In contrast, unhealthy neurons displayed phase-dark cell bodies, fragmented and beaded neurites, and condensed Hoechst-stained chromatin. Percentage survival equals the average number of healthy neurons across the four fields of view at the end of the treatment divided by the average number of healthy neurons before treatment multiplied by 100. Results correspond to the averages of four wells per condition from two independent experiments.

### Cell culture Western blottings

Following treatment, cells were rinsed with PBS and lysed in Laemmli sample buffer containing 5% β-mercaptoethanol. Proteins were separated on 12.5% SDS-PAGE gels and electroblotted onto supported nitrocellulose membranes. Membranes were blocked in 5% non-fat milk in TBST for 1 h at room temperature. Antibodies were diluted in either 2.5% non-fat milk or 5% bovine serum albumin in TBST and incubated with the membranes at 4°C overnight. The following antibodies and dilutions were used: JUN (1:1000, Cell Signaling, catalog #9165, RRID:AB_2130165), phosphorylated Ser63-JUN (1:1000, Cell Signaling, catalog #9261, RRID:AB_2130159), phosphorylated Ser73-JUN (1:1000, Cell Signaling, catalog #3270, RRID:AB_2129575), and tubulin (1:2000, Sigma, catalog #T5168, RRID:AB_477579). Membranes were washed three times in TBS with 0.05% or 0.01% Tween 20 before incubation with horseradish peroxidase-conjugated goat anti-rabbit or goat anti-mouse antibodies (Bio-Rad) for 1 h at room temperature. Membranes were washed three more times in TBST before being processed for chemiluminescence using Supersignal West Dura substrate (Pierce). Densitometry was performed on images obtained from scanned films using NIH ImageJ software.

### Experimental design and statistical analyses

Statistical analyses and plots were done using Excel (Microsoft) and Prism 7.3 (GraphPad) software. Experiments testing *in vivo* effects of URMC-099 and CLFB_1134_ were initially done with male mice, to reduce potential variability in hippocampal synaptic density due to estrous-related changes in females (Woolley et al., 1990). Subsequent treatment cohorts for behavioral testing included female mice as well, and *n* for each sex is reported for each experiment in the results. For single-sex *in vivo* and all *in vitro* experiments, comparisons between groups were done for normally-distributed continuous data by one-way ANOVA followed in the primary analysis by Sidak *post hoc* tests for hypothesis-driven pairwise comparisons (*in vivo* experiments: vehicle-treated EAE vs sham-immunized mice, and URMC-099 or CLFB_1134_ vs vehicle-treated mice; *in vitro* experiments: URMC-099- vs vehicle-treated cultures). Where other group comparisons were interesting, secondary analysis used Tukey *post hoc* tests for all pairwise comparisons. For the behavioral experiments, which included both male and female mice, we used two-way ANOVA with sex and treatment as independent factors; after results showed no significant effect of sex or sex-treatment interaction on behavioral test scores, *post hoc* pairwise comparisons were done as above on the treatment main effect. Non-parametric EAE scores were compared using the two-tailed Mann–Whitney *U* test for each day after disease onset. Significance was accepted at *p* < 0.05; *p* values (reported out to four decimal places or <0.0001) and sample sizes are reported in the text of the results or figure legends, and superscripts following each test in the results refer to the statistical table which reports mean differences with 95% confidence intervals for group comparisons (Table 1). All data in the results and figures are expressed as mean ± SEM.

**Table 1.** Statistical table

	Comparison	Data structure	Type of test	Mean diff. (95% CI)
a	PSD95, EAE-V versus Sham-V	Normal	One-way ANOVA, Sidak *post hoc*	–0.24 (–0.45 to –0.02)
b	PSD95, EAE-099 versus EAE-V	Normal	One-way ANOVA, Sidak *post hoc*	0.25 (0.01 to 0.48)
c	EAE score (IHC cohort), EAE-099 versus EAE-V	Non-parametric	Mann–Whitney *U* test	–0.5 (–2.5 to 0.5)^*^
d	EAE score (conditioning cohort), EAE-099 versus EAE-V	Non-parametric	Mann–Whitney *U* test	–1 (–1 to 0)^*^
e	Microglia SA:volume, EAE-V versus Sham-V	Normal	One-way ANOVA, Tukey *post hoc*	–0.98 (–1.54 to –0.43)
f	Microglia SA:volume, EAE-099 versus Sham-V	Normal	One-way ANOVA, Tukey *post hoc*	–1.09 (–1.64 to –0.53)
g	Microglia SA:volume, EAE-1134 versus Sham-V	Normal	One-way ANOVA, Tukey *post hoc*	–0.81 (–1.33 to –0.28)
h	Iba1, EAE-099 versus Sham-V	Normal	One-way ANOVA, Tukey *post hoc*	0.44 (0.05 to 0.82)
i	CD68, EAE-V versus Sham-V	Normal	One-way ANOVA, Tukey *post hoc*	0.73 (–0.26 to 1.71)
j	iNOS, EAE-V versus Sham-V	Normal	One-way ANOVA, Sidak *post hoc*	0.93 (0.29 to 1.57)
k	iNOS, EAE-099 versus EAE-V	Normal	One-way ANOVA, Sidak *post hoc*	–0.61 (–1.23 to –0.01)
l	FcyR1-CD64, EAE-V versus Sham-V	Normal	One-way ANOVA, Sidak *post hoc*	0.56 (0.16 to 0.95)
m	Pro-IL1β, EAE-V versus Sham-V	Normal	One-way ANOVA, Sidak *post hoc*	0.87 (–0.02 to 1.77)
n	CD86, EAE-099 versus EAE-V	Normal	One-way ANOVA, Sidak *post hoc*	–0.22 (–0.39 to –0.05)
o	Iba1, EAE-V versus Sham-V	Normal	One-way ANOVA, Tukey *post hoc*	5.69 (0.63 to 10.75)
p	Iba1, EAE-099 versus EAE-V	Normal	One-way ANOVA, Tukey *post hoc*	7.75 (3.27 to 12.24)
q	C1q, EAE-V versus Sham-V	Normal	One-way ANOVA, Tukey *post hoc*	2.63 (0.62 to 4.63)
r	C1q, EAE-099 versus Sham-V	Normal	One-way ANOVA, Tukey *post hoc*	3.01 (1.37 to 4.65)
s	Neuronal apoptosis, 100 nM 099 versus vehicle (24 h)	Normal	One-way ANOVA, Sidak *post hoc*	–37.0 (–23.2 to –50.8)
t	Neuronal apoptosis, 300 nM 099 versus vehicle (24 h)	Normal	One-way ANOVA, Sidak *post hoc*	–38.2 (–24.4 to –52.0)
u	Neuronal apoptosis, 100 nM 099 versus vehicle (48 h)	Normal	One-way ANOVA, Sidak *post hoc*	–40.8 (–27.0 to –54.6)
v	Neuronal apoptosis, 300 nM 099 versus vehicle (248 h)	Normal	One-way ANOVA, Sidak *post hoc*	–53.9 (–40.1 to –67.7)
w	P Ser 73 JUN, 099 versus vehicle (8h)	Normal	One-way ANOVA, Sidak *post hoc*	–0.80 (–0.14 to –1.45)
x	P Ser 73 JUN, 099 versus vehicle (12h)	Normal	One-way ANOVA, Sidak *post hoc*	–0.99 (–0.33 to –1.64)
y	Context Δ freeze, EAE-V versus Sham-V (treatment main effect)	Normal	Two-way ANOVA, Sidak *post hoc*	–0.33 (–0.61 to –0.05)
z	Context Δ freeze, EAE-099 versus EAE-V (treatment main effect)	Normal	Two-way ANOVA, Sidak *post hoc*	0.32 (0.02 to 0.62)

* Mann–Whitney *U* test results reported for days with greatest mean difference between group scores (IHC cohort: days 5–6; fear conditioning cohort: days 10–12).

## Results

### URMC-099 prevents excitatory synapse loss in EAE hippocampus

**Figure 1.**
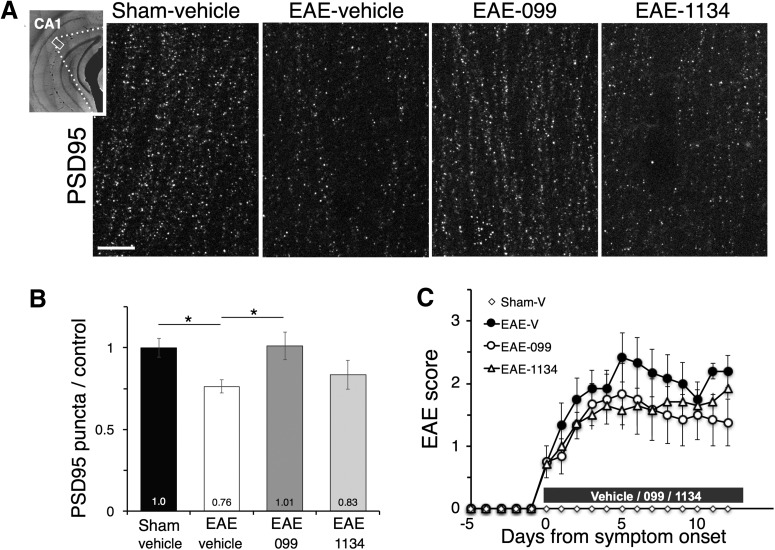
URMC-099 prevents excitatory synapse loss in EAE hippocampus. ***A***, Staining for PSD95-positive postsynaptic puncta in the stratum radiatum of hippocampal area CA1 (inset) was decreased in vehicle-treated EAE mice relative to sham-immunized controls. Treatment with URMC-099 beginning after the development of EAE motor deficits preserved synaptic puncta, while treatment with CLFB_1134_ did not. Scale bar: 10 µm. ***B***, Density of PSD95-positive puncta, quantified relative to sham-immunized controls; *p < 0.05, one-way ANOVA with Sidak *post hoc* test. ***C***, Treatment with 099 and 1134 in this cohort resulted in a modest reduction in mean severity of EAE motor deficits that was not significantly different from controls; *n* = 10 male mice (sham-vehicle), *n* = 6 male mice (EAE-vehicle and EAE-099), *n* = 7 male mice (EAE-1134). Data in all figures are expressed as mean ± SEM.

To test whether MLK3 inhibition could protect excitatory synapses in EAE hippocampus, we treated mice with URMC-099 or the highly-selective MLK3 inhibitor CLFB_1134_ beginning after the onset of EAE symptoms. URMC-099 treatment preserved excitatory synapses in hippocampal area CA1 of EAE mice (Fig. 1*A*,*B*): while PSD95-positive puncta counts were decreased in vehicle-treated EAE mice (*n* = 6 male mice) to 0.76 ± 0.04 relative to levels of sham-immunized controls (*n* = 10 male mice, *p* = 0.028, Sidak *post hoc* test^a^), URMC-099 restored counts to 1.01 ± 0.08 relative to controls (*n* = 6 male mice, *p* = 0.021 vs EAE-vehicle, Sidak *post hoc* test^b^). In contrast, CLFB_1134_ was not effective at protecting synapses, with hippocampal PSD95-positive puncta reduced to 0.83 ± 0.09 of control levels (*n* = 7 male mice), similar to vehicle-treated EAE mice. Neither drug prevented development of motor deficits in EAE, with each one causing modest reductions in EAE score that were not significant in this treatment cohort^c^ (Fig. 1*C*), and became statistically significant but less pronounced in a larger subsequent cohort treated with URMC-099 or vehicle^d^ (as discussed below, URMC-099 preserves hippocampal-dependent fear conditioning in EAE mice).

### URMC-099 modulates the phenotype of microglial activation in EAE hippocampus

**Figure 2.**
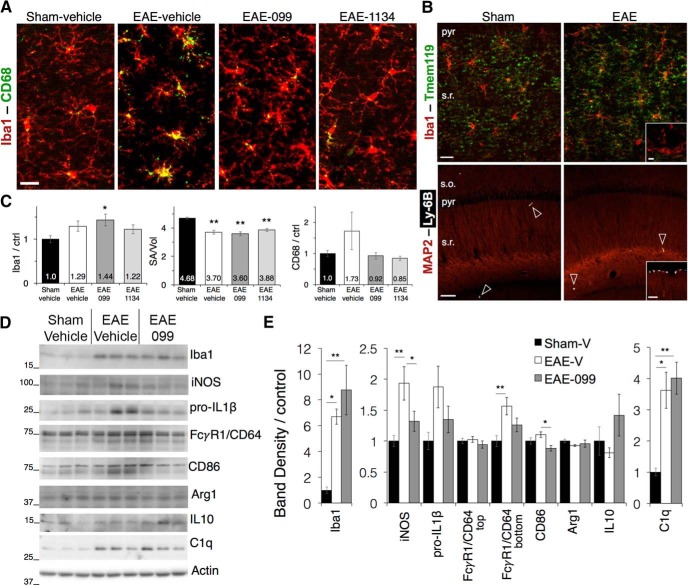
URMC-099 modulates the phenotype of activated microglia in EAE hippocampus. ***A***, Staining for Iba1 in hippocampal area CA1 shows ramified microglia in sham-immunized mice, and microglia with shorter, thicker processes in EAE. ***B***, Iba1-positive cells in the hippocampi of both sham and EAE mice coexpress microglia-specific marker Tmem119 along their processes and to a more variable extent in the cell body (upper panels). Iba1-positive, Tmem119-negative cells consistent with monocyte-derived macrophages could be found in the pia during EAE (inset), but not in or around the hippocampus. Staining for Ly-6B, expressed on neutrophils and some recently-generated macrophages, is sparse in sham and EAE hippocampi (lower panels, arrowheads), although clusters occurred sporadically in the pia and other area of some EAE brains (inset). Scale bars: 20 µm. s.o., CA1 stratum oriens; pyr, pyramidal layer; s.r., stratum radiatum. ***C***, Quantification of microglial images shows increased Iba1 intensity and reduction in the ratio of surface area to volume in EAE microglia regardless of treatment with URMC-099 or CLFB_1134_. Microglial expression of lysosomal marker CD68 is increased in many hippocampi from vehicle-treated EAE mice but not in mice treated with URMC-099 or CLFB_1134_; *n* = 10 male mice (sham-vehicle), *n* = 6 male mice (EAE-vehicle and EAE-099), *n* = 7 male mice (EAE-1134). ***D***, ***E***, Western blottings and band densitometry for markers of inflammation and microglial phenotype in hippocampal protein extracts. Markers associated with proinflammatory microglial activation tended to increase in vehicle-treated EAE hippocampi and were decreased by URMC-099, with significant changes in iNOS (inducible nitric oxide synthase) and immunoglobulin receptor FCγR1/CD64 (lower single band), and a non-significant trend toward increased pro-IL1β. Proinflammatory marker CD86 does not significantly increase in vehicle-treated EAE but is decreased by URMC-099 treatment. Arg1 and IL-10, canonical markers of anti-inflammatory alternative activation, are not significantly changed in EAE or with URMC-099. Consistent with immunostaining results, Iba1 is up-regulated in vehicle-treated EAE hippocampi and is further increased with URMC-099 treatment. Complement component C1q follows a similar pattern; *n* = 10 male mice (sham-vehicle), *n* = 7 male mice (EAE-vehicle), *n* = 11 male mice (EAE-099); one representative blot with three lanes per condition is depicted; *p < 0.05, **p < 0.01, one-way ANOVA with Sidak (iNOS, FCγR1/CD64, CD86) or Tukey (Iba1, SA/Vol, C1q) *post hoc* test.

Microglial immunostaining in hippocampal area CA1 showed increased Iba1 expression and a shift from a ramified morphology with thin processes to a more amoeboid morphology with thicker, shorter processes consistent with activation in EAE (Fig. 2*A*). Coexpression of Tmem119 along processes and/or cell bodies (Fig. 2*B*) suggests that Iba1-positive cells in the hippocampus represented brain-resident microglia rather than monocyte-derived macrophages (Bennett et al., 2016), and staining for Ly-6B, expressed by neutrophils and some recently-generated macrophages (Rosas et al., 2010) showed that infiltrating myeloid cells were sparse in the hippocampus regardless of treatment condition (1.85 ± 0.19 cells per 10× field in *n* = 5 male sham control mice, vs 2.44 ± 1.12 in *n* = 4 male EAE-vehicle mice, and 2.05 ± 0.76 in *n* = 5 male EAE-099 mice, one-way ANOVA *F*_(2,11)_ = 0.16, *p* = 0.86), although sporadic clusters could be found in the pial meningeal layer in EAE (Fig. 2*B*, insets).

Microglial morphologic changes were reflected by a decrease in the ratio of microglial surface area to volume in vehicle-treated EAE mice (*n* = 6 male mice) and were not prevented by treatment with URMC-099 (*n* = 6 male mice) or CLFB_1134_ (Fig. 2*C*; *n* = 7 male mice, *p* = 0.003 for EAE-vehicle vs sham control^e^, *p* < 0.0001 for EAE-099 vs sham control^f^, and *p* = 0.002 for EAE-1134 vs sham control^g^, Tukey *post hoc* test). Increases in microglial Iba1 were highest and reached significance only in URMC-099-treated EAE mice (*p* = 0.023 vs sham control, Tukey *post hoc* test^h^). Microglial expression of CD68, a lysosomal marker associated with phagocytosis during activation, was increased in many hippocampal imaging fields in vehicle-treated EAE mice, although the extent was widely variable and quantification did not reach statistical significance^i^. URMC-099 and CLFB_1134_ both prevented any increases in microglial CD68 expression, suggesting that while neither agent restores microglia to a resting morphology in the EAE hippocampus, they may modulate their activation state.

We further evaluated microglial phenotypic markers in hippocampal protein extracts from mice treated with URMC-099 or vehicle (Fig. 2*D*,*E*). Markers canonically associated with proinflammatory microglial activation tended to be increased in vehicle-treated EAE (*n* = 7 male mice) compared to sham-immunized controls (*n* = 10 male mice) and attenuated by URMC-099 (*n* = 11 male mice), with statistically significant changes in inducible nitric oxide synthase (iNOS; *p* = 0.003 EAE-vehicle vs sham^j^ and *p =* 0.04 EAE-vehicle vs EAE-URMC-099^k^, one-way ANOVA with Sidak *post hoc* test) and immunoglobulin receptor FCγR1/CD64 (lower single band; *p* = 0.004 EAE-vehicle vs sham, Sidak *post hoc* test^l^), and a non-significant trend toward increased pro-IL1β (*p* = 0.06 EAE-vehicle vs sham, Sidak *post hoc* test^m^). Interestingly, the costimulatory molecule CD86, expressed by antigen-presenting cells as well as macrophages, increased little in vehicle-treated EAE but was significantly decreased by URMC-099 treatment (*p* = 0.011 EAE-vehicle vs EAE-URMC-099, Sidak *post hoc* test^n^). Arg1 and IL10, markers associated with anti-inflammatory alternative activation, were not significantly changed in EAE or with URMC-099. Consistent with immunostaining results, Iba1 expression was increased in EAE hippocampi during both vehicle and URMC-099 treatment (*p* = 0.025 EAE-vehicle vs sham^o^, and *p* = 0.0006 EAE-URMC-099 vs sham^p^, one-way ANOVA with Tukey *post hoc* test). Expression of complement component C1q, which is increased in neurons in MS gray matter and is thought to be associated with synapse elimination, followed a similar pattern (*p* = 0.01 EAE-vehicle vs sham^q^ and *p* = 0.0006 EAE-URMC-099 vs sham^r^, Tukey *post hoc* test) that did not correlate with URMC-099’s synaptic protection. In aggregate, these results suggest that URMC-099 does not prevent microglia from becoming activated in EAE hippocampi but does seem to shift them toward a less inflammatory phenotype that may contribute to synaptic preservation.

### URMC-099 prevents neuronal apoptosis *in vitro*

**Figure 3.**
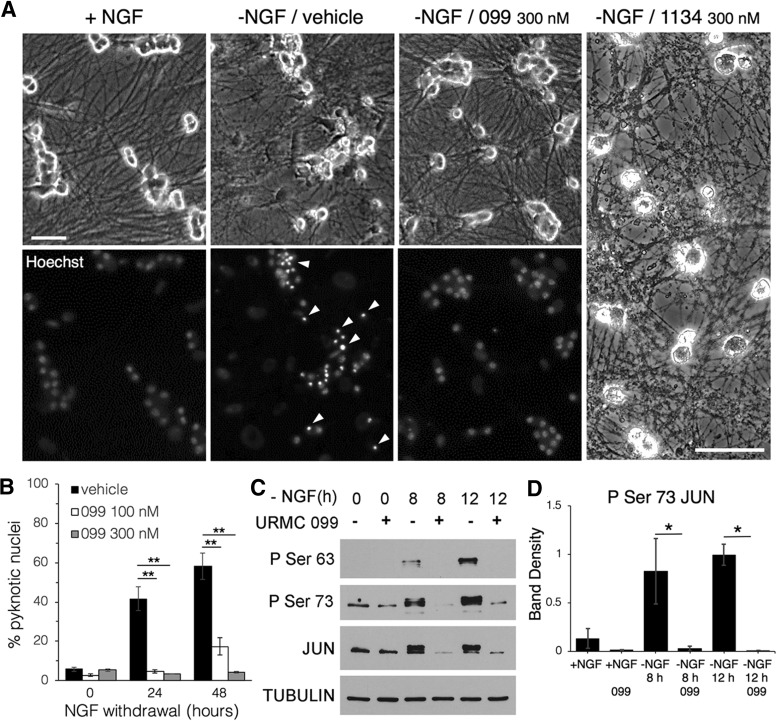
URMC-099 protects neuronal cultures during growth factor deprivation. ***A***, Phase-contrast images (upper) of cultured SCG neurons show disruption of neurite integrity after 48 h of NGF deprivation, and Hoechst DNA staining (lower) shows condensed pyknotic nuclei (arrowheads indicate examples) in the same neurons. Cultures treated with URMC-099 (300 nM) maintained neurite integrity and diffuse nuclear chromatin staining indicative of healthy neurons after 48 h of NGF deprivation. Cultures treated with CLFB_1134_ had marked fragmentation of neurites, and cellular damage severe enough to preclude Hoechst analysis. Scale bars: 60 µm. ***B***, Quantification of dead SCGs identified by pyknotic nuclei shows a dose-dependent protective effect for URMC-099 following NGF deprivation (*n* = 4 cultures for each condition). ***C***, Western blottings for JUN, which mediates SCG apoptosis during NGF withdrawal and is activated downstream of MLK signaling via phosphorylation by JNK, show increased JUN expression and phosphorylation at serine residues 63 and 73 in protein isolates from SCG cultures following NGF withdrawal, which is nearly abolished by treatment with URMC-099 (300 nM, *n* = 3 experiments); *p < 0.05, **p < 0.0001, one-way ANOVA with Sidak *post hoc* test.

Given the efficacy of URMC-099 but not CLFB_1134_ at preserving hippocampal synapses in the face of microglial activation in EAE, we asked whether the drugs differ in their ability to exert direct protective effects on neurons. As direct versus glia-mediated neuroprotection is difficult to differentiate *in vivo*, we tested both drugs in a culture system in which MLK signaling can drive neuronal apoptosis and rescue by broad-spectrum MLK inhibitors has been firmly established (Xu et al., 2001; Harris et al., 2002). Sympathetic neurons cultured from murine SCG degenerate after withdrawal of NGF from the culture media, with fragmentation of neurites and condensation of chromatin in pyknotic nuclei (Fig. 3*A*). Addition of 100 or 300 nM URMC-099 prevented neuronal death and maintained healthy neurites up to 48 h after NGF withdrawal in a dose-dependent manner (Fig. 3*A*,*B*; *n* = 4 cultures per condition, *p* < 0.0001 for each URMC-099 dose vs vehicle at 24 and 48 h, one-way ANOVA with Sidak *post hoc* test^s–v^). In similar cultures, we evaluated expression and phosphorylation of JUN, a target of the JNK pathway that mediates apoptosis during NGF withdrawal and can be activated by MLKs and likely other kinases that mediate proinflammatory signaling such as LRRK2 (Puccini et al., 2015). URMC-099 (300 nM) blocked increases in JUN expression as well as phosphorylation at serine residues 63 and 73 (Fig. 3*C*,*D*; *n* = 3 cultures per condition, *p* = 0.015 for P Ser 73 JUN in 099- vs vehicle-treated cultures at 8 h and *p* = 0.003 at 12 h, one-way ANOVA with Sidak *post hoc* test^w,x^), consistent with reduced JNK activity downstream of MLK inhibition. Treatment with CLFB_1134_ failed to provide protection or trophic support to NGF-deprived neurons, which underwent neuritic beading and degeneration of cell bodies to an extent that precluded quantification of pyknotic versus healthy nuclei, suggesting that the neuroprotective effects of broad-spectrum MLK inhibition *in vitro,* like *in vivo,* are not reproduced by an inhibitor that is highly selective for MLK3. Thus, subsequent experiments focused on further characterizing the effects of URMC-099 in EAE hippocampus.

### URMC-099 preserves hippocampal-dependent fear conditioning in EAE mice

**Figure 4.**
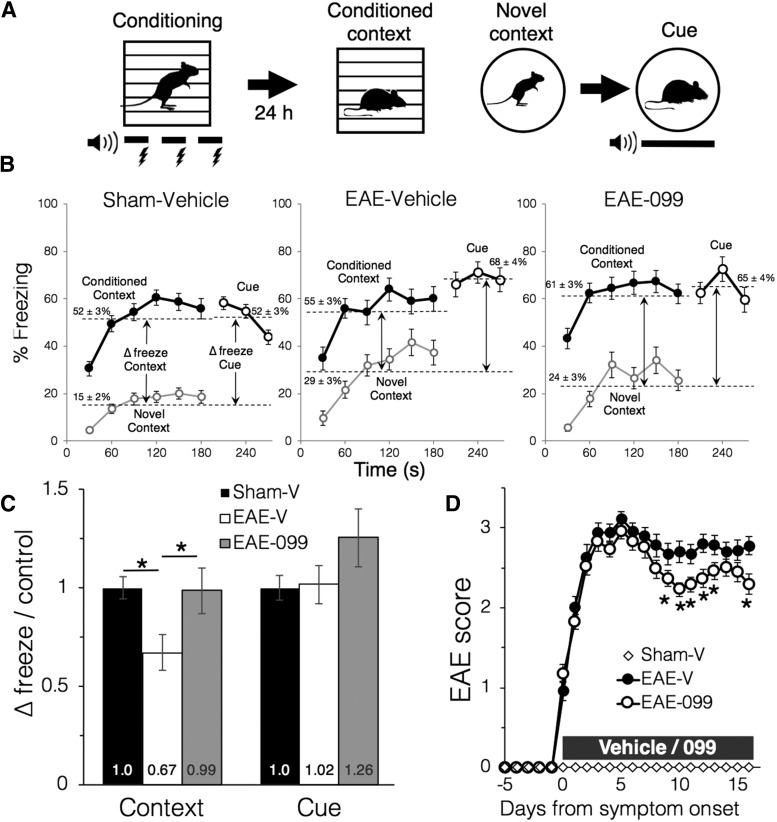
URMC-099 preserves contextual fear conditioning in EAE mice. **A.** Contextual and cued fear conditioning protocol for EAE mice. Mice were conditioned in an isolation chamber where they were presented three times with an auditory cue paired with a foot shock; 24 h later, mice were evaluated for episodes of freezing after being returned to the identical chamber (conditioned context), introduced to a chamber modified in shape, texture and odor (novel context), and presented with the auditory cue (cue). ***B***, Group averages of freezing responses during each of the three tests, expressed as % of time spent freezing in 30-s epochs. Mean rates of freezing (dotted lines) increased in the conditioned context and with cue presentation relative to the unconditioned novel context in all groups, although EAE mice with motor deficits had higher rates of immobility in the novel context compared to sham-immunized controls. Mean freezing rates in the novel context were subtracted from those in the conditioned context and during cue presentation (Δ freeze) to differentiate freezing due to conditioning from non-specific immobility. ***C***, Δ freeze values show that deficits in contextual fear conditioning in vehicle-treated EAE mice were restored to control levels by URMC-099 treatment. EAE mice showed no deficits in auditory cue conditioning, with similar Δ freeze values in all groups; *p < 0.05, two-way ANOVA with Sidak *post hoc* test of the treatment main effect. ***D***, EAE scores for the mouse cohorts that underwent behavioral testing show that URMC-099 treatment after symptom onset did not affect peak EAE severity and was associated with modest recovery that was statistically different from vehicle-treated mice after 9–13 d of disease; *p < 0.5, Mann–Whitney *U* test. For all panels, *n* = 29 mice (sham-vehicle), *n* = 18 mice (EAE-vehicle), *n* = 21 mice (EAE-099).

We tested whether URMC-099 could preserve hippocampal-dependent learning and memory in EAE mice, and chose contextual fear conditioning because it is less dependent on locomotion than maze or exploratory learning paradigms and could be adapted to mice with motor deficits. Furthermore, pairing hippocampal-dependent contextual conditioning with a test of cued conditioning which is independent of the hippocampus (Phillips and LeDoux, 1992) but shares the same output measures provided a positive control to test whether conditioning effects could be detected in the presence of EAE motor deficits. Because EAE mice had greater rates of immobility at baseline and during testing in an unconditioned novel context that was not specific to conditioned stimuli (Fig. 4*B*), we quantified the increase in freezing behavior due to context or cued conditioning as the difference between the percentage of time spent freezing during the conditioned context test and the novel context test (context Δ freeze), and between the cue presentation and the novel context test (cue Δ freeze). This provided a measure of the mouse’s response to the conditioned context and cue while minimizing confound due to motor deficits or reduced exploratory behavior associated with EAE. Cohorts for behavioral testing included both male and female mice, although two-way ANOVA showed no significant effect of sex or of the interaction between sex and treatment on behavioral scores (*F*_(1,62)_ = 0.40, *p* = 0.53 for sex main effect and *F*_(2,62)_ = 1.10, *p* = 0.34 for interaction effect on context Δ freeze) and further analysis focused on treatment main effects.

Vehicle-treated EAE mice (*n* = 15 male and *n* = 3 female mice) showed a decrement in contextual fear conditioning (Fig. 4*C*), with context Δ freeze reduced to 0.67 ± 0.09 relative to sham-immunized controls (*n* = 24 male and *n* = 5 female mice, *p* = 0.019, two-way ANOVA with Sidak *post hoc* test on the treatment main effect^y^). Freezing responses to the conditioned context were restored to control levels in URMC-099-treated EAE mice (0.99 ± 0.12, *n* = 18 male and *n* = 3 female mice), a significant improvement compared to vehicle-treated EAE mice (*p* = 0.038, Sidak *post hoc* test^z^). Both vehicle- and URMC-099-treated EAE mice had similar increases in freezing as control mice during presentation of the auditory cue (two-way ANOVA, treatment main effect, *F*_(2,62)_ = 0.415, *p* = 0.66), suggesting that EAE deficits do not limit the sensitivity of the test to detect fear conditioning responses. EAE scores from the mouse cohorts that underwent fear conditioning (Fig. 4*D*) show that URMC-099- and vehicle-treated EAE mice had similar severity of motor deficits at the peak of disease, while URMC-099-treated mice recovered to slightly less severe scores that were statistically significant from 9 to 13 d after disease onset (*p* < 0.05, Mann–Whitney *U* test^d^).

## Discussion

These results demonstrate that the kinase inhibitor URMC-099 can protect hippocampal synapses and maintain hippocampal-dependent learning and memory in an EAE model that recapitulates the excitatory synaptic injury that occurs widely within the gray matter in MS. We have previously described URMC-099 as a broad-spectrum MLK3 inhibitor (Goodfellow et al., 2013) because of its ability to modulate multiple kinase “control hubs” related to restoration of the balance between innate immune cells and end-organ target cells such as neurons (Marker et al., 2013), especially with respect to subcellular synaptic elements. URMC-099 appears to modulate activated microglia in the EAE hippocampus toward a less inflammatory phenotype and may have direct protective effects on neurons as well. URMC-099 has similarly reduced markers of proinflammatory microglial activation in mouse models of Alzheimer’s disease (Kiyota et al., 2018) and HIV-associated neuroinflammation (Marker et al., 2013), suggesting that it may have potential to shift microglia toward neuroprotective phenotypes in a variety of neuroinflammatory conditions.

In contrast to other treatments that have shown neuroprotective effects in EAE gray matter when begun before disease onset (Centonze et al., 2009; Kim et al., 2012; Bellizzi et al., 2016; Di Filippo et al., 2016), URMC-099 protected the hippocampus when administered after the onset of EAE motor deficits. At that point in this model, immune activation and infiltration into the CNS is at or near its peak (Murphy et al., 2010) and deficits in hippocampal-dependent learning and memory are already established (Kim et al., 2012). URMC-099 treatment had no effect on the peak severity of EAE motor deficits, which reflect damage due to lymphocyte-driven inflammation in the spinal cord, and did not prevent microglia in the hippocampus from adopting an activated morphology. This suggests that URMC-099 treatment after disease onset did not prevent inflammation in the acute phase of EAE, but was subsequently able to provide hippocampal neuroprotection in the chronic phase.

Microglial activation is the predominant immunopathology in the hippocampus in the chronic phase of this EAE model; prior studies have found no increase in lymphocytes (Ziehn et al., 2010) and we show no change in the sparse peripheral myeloid cells in the hippocampus of EAE versus sham-immunized mice. Converging evidence from multiple studies have established that proinflammatory microglia can disrupt synaptic structure and function in EAE gray matter via production of inflammatory signaling molecules including IL-1β, TNFα, PAF, reactive oxygen species, and nitric oxide (Centonze et al., 2009; Kim et al., 2012; Rossi et al., 2012; Bellizzi et al., 2016; Di Filippo et al., 2016; Mancini et al., 2018). URMC-099 reduced inducible nitric oxide synthase and other markers of proinflammatory microglial activation in the EAE hippocampus, appearing to shift microglia toward a less toxic and perhaps more protective phenotype. Microglial can adopt a diverse range of activation states that can shift during the course of disease and have potential to be harmful or protective depending on their context (Lewis et al., 2014; Ajami et al., 2018; Ayata et al., 2018), and how URMC-099 may modulate these states for neuroprotection needs further study. We propose that the ability to shift already-activated microglia from toxic to protective states may be particularly important for neuroprotection in MS, in which gray matter injury and cognitive dysfunction can begin at the earliest disease stages, are chronically associated with local microglial activation, and almost inevitably progress despite current immunomodulatory therapies (Amato et al., 2004; Dalton et al., 2004; Magliozzi et al., 2010; Weinstock-Guttman et al., 2012).

URMC-099 was more effective at protecting synapses *in vivo* than the highly-selective MLK3 inhibitor CLFB_1134_, suggesting that its broader range of kinase inhibition is necessary for its neuroprotective effects. Furthermore, CLFB_1134_ failed to match URMC-099’s ability to protect cultured sympathetic neurons during NGF withdrawal, a model system in which signaling by MLKs and possibly other kinases drives apoptosis via downstream JNK activation and in which a variety of approaches to MLK inhibition, including introduction of dominant-negative MLK3 isoforms and other MLK inhibitors with broad specificity, have been well established as protective (Xu et al., 2001; Harris et al., 2002; Goodfellow et al., 2013). These data demonstrate that highly selective MLK3 inhibition is less effective for neuroprotection and potential neurorestoration after the onset of disease than broader inhibition of a combination of kinases. This is not surprising given that inflammation and cell stress can activate multiple MLK family members in the brain as well as other kinases targeted by URMC-099, including LRRK2 that can contribute to proinflammatory microglial activation and production of signaling molecules with neurotoxic potential (Moehle et al., 2012; Puccini et al., 2015). These data suggest that overlap and/or synergies among the multiple targets of URMC-099’s kinase inhibition is critical for its neuroprotective properties.

URMC-099 has been effective and well tolerated in a broad variety of *in vivo* models of degenerative diseases associated with inflammation including HIV-1 associated neurologic disease, Alzheimer’s disease, as well as inflammatory liver injury in non-alcoholic steatohepatitis (Marker et al., 2013; Dong et al., 2016; Tomita et al., 2017). While activity against multiple kinases does raise concern for increasing risk of adverse effects, we propose that evaluating for such effects directly may be more fruitful for therapeutic development than holding out hope that more selective targeting of a single kinase may be as effective. URMC-099’s exemplary safety profile in the above *in vivo* models, and the fact that the previous non-selective MLK inhibitor CEP-1347 was well tolerated without significant adverse effects compared to placebo in clinical trials (Investigators PSGP, 2007; Ma et al., 2013), further strengthens the argument for advancing this therapeutic approach toward clinical applications.

The combination of URMC-099’s anti-inflammatory and neuroprotective effects was successful at protecting synaptic structures while maintaining synaptic function in EAE hippocampus. Achieving both is necessary for treatment of a chronic neurodegenerative disease, but can be difficult given that many inflammatory signaling molecules with neurotoxic effects including IL-1β, TNFα, PAF, reactive oxygen species, and nitric oxide also play critical roles in physiologic synaptic plasticity (O'Dell et al., 1991; Kato et al., 1994; Klann, 1998; Schneider et al., 1998; Stellwagen and Malenka, 2006). The therapeutic window for targeting signaling at the synapse can be almost impossibly narrow in neuroinflammatory disease: while blocking pathologic versus physiologic NMDA receptor activation has been considered promising for multiple neurodegenerative diseases (Lipton and Rosenberg, 1994), we have shown that similar activation can promote physiologic plasticity or excitotoxic injury depending on the presence of inflammatory mediators (Bellizzi et al., 2005). Indeed, attempts to treat MS patients with the uncompetitive NMDA receptor blocker memantine have resulted in no improvement or even worsening of neurologic deficits (Villoslada et al., 2009; Lovera et al., 2010). Likewise, PAF receptor antagonists can protect synaptic structures in EAE (Bellizzi et al., 2016) but also inhibit performance on tasks of learning and memory (Jerusalinsky et al., 1994). Thus, treatments like URMC-099 that can shift the inflammatory milieu and provide neuroprotection upstream of synaptic activation may hold more promise for restoration of gray matter function in MS and other neurodegenerative diseases.

## Synthesis

Reviewing Editor: Liisa Galea, University of British Columbia Decisions are customarily a result of the Reviewing Editor and the peer reviewers coming together and discussing their recommendations until a consensus is reached. When revisions are invited, a fact-based synthesis statement explaining their decision and outlining what is needed to prepare a revision will be listed below. The following reviewer(s) agreed to reveal their identity: David Pleasure, Paolo Calabresi. Note: If this manuscript was transferred from JNeurosci and a decision was made to accept the manuscript without peer review, a brief statement to this effect will instead be what is listed below.

In their manuscript, “The mixed-lineage kinase inhibitor URMC-099 protects hippocampal synapses in experimental autoimmune encephalomyelitis”, Bellizzi and colleagues investigated the possible neuroprotective effects of a mixed lineage kinase (MLK) inhibitor (URMC-099), developed by their research group, in an experimental model of MS. In particular, they found that URMC-099, a broad spectrum MLK inhibitor with additional activity against LRRK2 and other kinases, prevented loss of postsynaptic structures in the hippocampus and reversed deficits in hippocampal-dependent contextual fear conditioning in mice affected by EAE mice when administered after the onset of motor symptoms.

All of the reviewers agree that this was a well written manuscript with important implications suggesting potential neuroprotective strategies for MS.

As suggested in the prior review, it would have improved the manuscript if an in vitro dose-response study could help in establishing the neuroprotective effect of the drug but the reviewers are not willing to make this a requirement of acceptance.

The microglial modulation exerted by URMC-099 and CLFB-1134 is not clear at all. Authors stated that “URMC-099 and CLFB-1134 both prevented any increases in microglial CD68 expression, suggesting that treatment with either agent does not block activation of microglia in the EAE hippocampus but may modulate the biologic response of their activated phenotype”. The potential role of persistent microglial activation in the neuro-inflammatory related neurodegeneration has been largely characterized. The fact that microglial activation has not been significantly altered by URMC-099 and CLFB-1134 administration potentially reduces the impact of the work. Interestingly, authors found that URMC-099 significantly reduced the expression of CD68, iNOS and FCγR1/CD64, with a non-significant reduction of IL-1β. Considering these results, authors could take advantage in discussing some works showing the relationship between microglial activation and the activation of NO-pathway in enhancing neuronal mitochondrial dysfunction during EAE (Mancini et al., Neurobiol Dis, 2018), or the relationship existing between hippocampal microglial CD68 expression and the disruption of hippocampal synaptic plasticity and hippocampal-dependent memory during EAE (Di Filippo et al., Sci Rep, 2016).

In their rebuttal and manuscript, the authors overstate the report by Ziehn et al (2010) that there are no inflammatory cells in hippocampus in this model. In fact, Ziehn et al made no effort to discriminate between activated microglia and monocyte-derived macrophages. Further, they did not exclude an early invasion by neutrophils. Immunohistologiccal reagents are now available that can help with this discrimination (though FACS, of course, would be more definitive).

The authors used both sexes but did not analyze by sex and this is a concern as there are noted sex differences in EAE susceptibility (although apparently not in C57BL6 but this should be noted) but there are sex differences in fear conditioning, MS, and immune signalling (i.e. The importance of studying sex differences in disease: The example of multiple sclerosis. Golden LC, Voskuhl R. J Neurosci Res. 2017 Jan 2;95(1-2):633-643; Sex effects on inflammatory and neurodegenerative processes in multiple sclerosis. Ramien C, Taenzer A, Lupu A, Heckmann N, Engler JB, Patas K, Friese MA, Gold SM. Neurosci Biobehav Rev. 2016 Aug;67:137-46; The influence of sex hormones on cytokines in multiple sclerosis and experimental autoimmune encephalomyelitis: a review. van den Broek HH, Damoiseaux JG, De Baets MH, Hupperts RM. Mult Scler. 2005 Jun;11(3):349-59). The advice here would be to use sex as a factor in the analyses as at the very least a covariate but preferably an independent factor. It is possible that the use of sex as a factor in the analyses will provide some insight into the larger errors on some measures. In addition, the sex ratio per study should be given as it is not clear if an equal number of males and females were used.

Minor

Please remove the word “gender” from line 157 and replace it with “sex” as gender is a psychosocial construct (roles on which a given society deems appropriate for men or women) and not appropriate in animal modelling.
